# Impact of teneligliptin on oxidative stress and endothelial function in type 2 diabetes patients with chronic kidney disease: a case–control study

**DOI:** 10.1186/s12933-016-0396-3

**Published:** 2016-05-17

**Authors:** Masaaki Sagara, Kunihiro Suzuki, Chie Aoki, Seiichi Tanaka, Isao Taguchi, Teruo Inoue, Yoshimasa Aso

**Affiliations:** Department of Endocrinology and Metabolism, Dokkyo Medical University, 880 Kitakobayashi, Mibu, Shimotsugagun, Tochigi 321-0293 Japan; Department of Cardiology, Dokkyo Medical University, Koshigaya, Saitama Japan; Department of Cardiovascular Medicine, Dokkyo Medical University, Mibu, Tochigi Japan

**Keywords:** Teneligliptin, Sitagliptin, Chronic kidney disease, Endothelial function, Oxidative stress

## Abstract

**Background:**

The aim of the present study was to elucidate the effect of teneligliptin on oxidative stress and endothelial function in Japanese patients with type 2 diabetes and chronic kidney disease (CKD).

**Methods:**

Forty-five patients with type 2 diabetes and CKD who received sitagliptin for at least 12 months were randomized to either continue sitagliptin (n = 23) or switch to teneligliptin (n = 22) for 24 weeks. The following parameters were evaluated at baseline and after 24 weeks of treatment with continued sitagliptin or teneligliptin: blood pressure, haemoglobin A1c (HbA1c), estimated glomerular filtration rate (eGFR), urinary albumin excretion, endothelial function by reactive hyperaemia index (RHI; EndoPAT^®^ system), reactive oxygen metabolites (ROMs) measured by the d-ROMS test, 8-hydroxy-2′-deoxyguanosine, urinary liver-type fatty acid binding protein (L-FABP), and urinary 8-isoprostane.

**Results:**

The two groups did not significantly differ with regard to age, male-to-female ratio, duration of diabetes, body mass index, HbA1c, eGFR, or urinary albumin excretion levels at baseline. We found no significant differences in changes of HbA1c, eGFR, or urinary albumin excretion levels between the two groups after 24 weeks of treatment. However, treatment with teneligliptin, but not sitagliptin, significantly improved RHI values and was correlated with the percent changes in RHI and d-ROMs.

**Conclusions:**

The present study demonstrated that teneligliptin, can improve endothelial function and reduce renal and vascular oxidative stress in patients with type 2 diabetes and CKD, independently of reducing albuminuria or improving glucose control.

*Trial registration* UMIN000017180

## Background

The prevalence of type 2 diabetes is increasing rapidly worldwide, affecting an estimated 285 million patients in 2010 and expected to reach 439 million by 2030 [1]. This increase has also been seen in Japan, where the majority of the 7.2 million people with diabetes mellitus are aged between 20 and 79, and the number of people with diabetes mellitus will increase to 10.15 million by 2030 [2, 3]. Chronic kidney disease (CKD) is common in patients with type 2 diabetes mellitus, as well as ischaemic heart disease and stroke [4]. In addition, Alan et al. reported that CKD contributes to the development of cardiovascular diseases, and thus it increases the risk of death and cardiovascular events [5].

It has been reported that approximately 40 % of patients with type 2 diabetes mellitus have elevated urinary albumin excretion consistent with underlying renal disease, and 17 % of patients with diabetes have CKD [6]. Intensive glycaemic control in type 2 diabetes mellitus significantly reduced the primary composite outcome of microvascular events, mainly as a consequence of a reduction in nephropathy [7]. However, options for anti-hyperglycaemic agents in such patients are limited due to safety and tolerability concerns. Metformin, sulfonylureas, and thiazolidinedione are associated with an increased incidence of hypoglycaemia, weight gain, and lactic acidosis in patients with type 2 diabetes and CKD [8]. Therefore, many patients with type 2 diabetes and CKD do not achieve or maintain adequate glycaemic control.

Teneligliptin, a novel dipeptidyl peptidase-4 (DPP-4) inhibitor, is substantially metabolized in the liver, and its serum concentration is not affected by even severe renal impairment [9]. By contrast, sitagliptin is cleared primarily by the kidney, with 80 % of oral doses excreted unchanged in the urine [6, 10]. Based on its renal clearance, patients with moderate and severe renal insufficiency should receive one-half of the usual clinical dose (50 or 25 mg daily in Japan) [11]. In the context of pharmacological characteristics, teneligliptin is advantageous in that the dose remains the same independent of renal function [8, 12].

There have been no reports on the effects of teneligliptin compared to sitagliptin in terms of protective effects on vascular endothelial function, suppression of oxidative stress, and effect on biomarkers of tubulointerstitial kidney damage such as liver fatty acid-binding protein (L-FABP) [13] in patients with type 2 diabetes and CKD. In the present study, we aimed to investigate the effect of switching from teneligliptin to sitagliptin for 24 weeks on endothelial function, oxidative stress markers, and biomarkers of tubulointerstitial kidney damage.

## Methods

### Subjects

Forty-five patients with type 2 diabetes and CKD who received sitagliptin for at least 12 months were randomized to either continue sitagliptin (n = 23) or switch to teneligliptin (n = 22) for 24 weeks. All patients had visited the Department of Endocrinology and Metabolism, Dokkyo Medical University Hospital on an outpatient basis for glycaemic control. The entry criteria included (1) age >20 years old, (2) type 2 diabetes with a haemoglobin A1c (HbA1c) >6.5 %, (3) CKD [estimated glomerular filtration rate (eGFR) <60 mL/min/1.73 m^2^ or microalbuminuria >30 mg/g Cr] [14], and (4) treatment with sitagliptin for 1 year or longer. The exclusion criteria were (1) type 1 diabetes, (2) severe diabetic complications such as ketoacidosis, (3) liver dysfunction, (4) pregnant or nursing women and those who might be pregnant, (5) chronic heart failure, (6) haemodialysis, (7) a history of stroke and cardiovascular events, and (8) any patient whom the investigator judged to be inappropriate for this study. Patients were given detailed explanations of the study protocol. Informed consent was obtained from each patient. The study protocol was approved by the Ethical Committee of Dokkyo Medical University. The trial was registered with the University Hospital Medical Information Network (UMIN No. #000017180).

### Study protocol

**Fig. 1:**
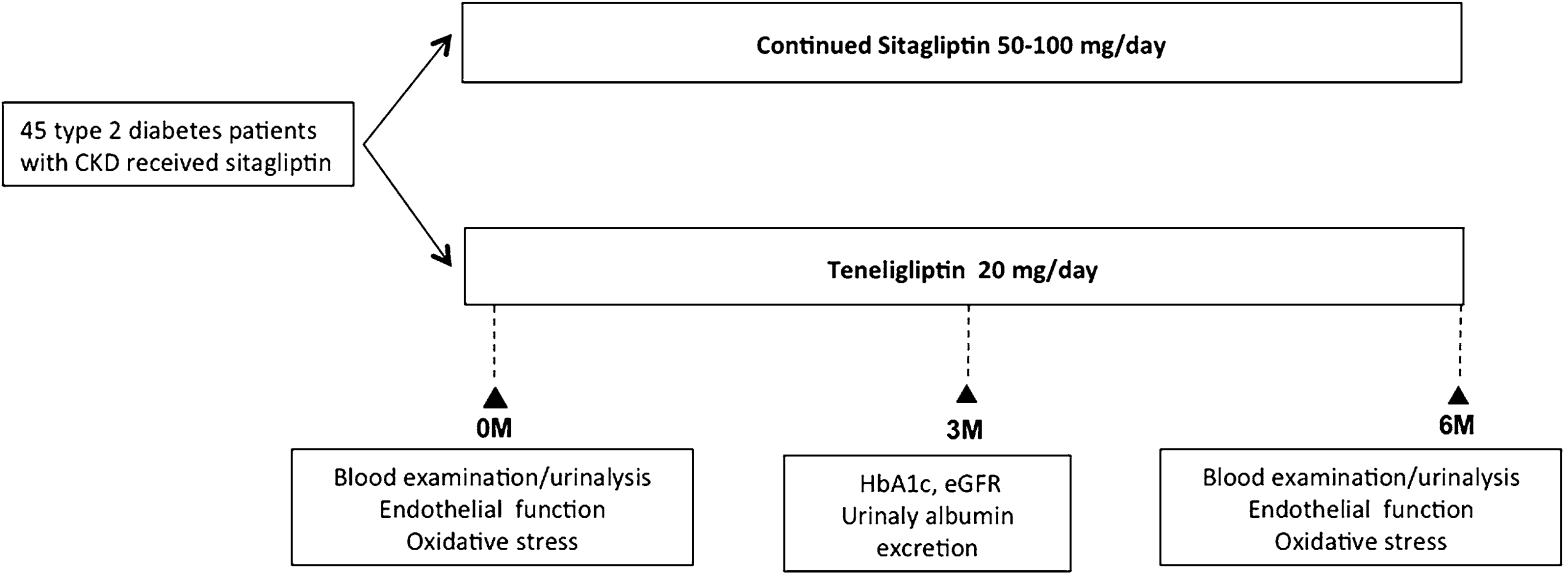
Study protocol. After outpatients received sitagliptin treatment for a period of 12 months or longer, they were randomized to the sitagliptin group, who continued to be treated with sitagliptin, or the teneligliptin group, whose treatment was switched to teneligliptin at the beginning of the study period

Figure 1 shows a summary of the study protocol. The present study was an open-label, prospective, randomized study. Patients were randomly divided into two groups: the sitagliptin group continued treatment with sitagliptin and the teneligliptin group switched treatment to teneligliptin at the beginning of the study period. Patients in both groups were assessed for the following parameters before the start of the study and 24 weeks after the continuation of sitagliptin or switch to teneligliptin. No changes were made to the type and dose of glucose-lowering drugs, angiotensin converting enzyme inhibitors, or angiotensin receptor blockers during the study period to avoid possible influences on endothelial function, production of oxidative stress, and blood pressure. These drugs had been prescribed for at least 12 months before the study.

### Assessment of endothelial function

Measurement of peripheral vasodilator response with a fingertip pulse amplitude tonometry (PAT) device is emerging as a useful method to assess vascular function [15].

Peripheral endothelial function was assessed by reactive hyperaemia peripheral arterial tonometry (RH-PAT) using an EndoPAT2000 device (Itamar Medical, Caesarea, Israel) as described previously [16]. Briefly, a blood pressure cuff was placed on 1 upper arm, while the contralateral arm served as a control. PAT probes were placed on 1 finger of each hand. After a 5-min equilibration period, the cuff was inflated to 60 mmHg above the systolic pressure (if systolic blood pressure was >140 mmHg) or to 200 mmHg (if systolic blood pressure was ≤140 mmHg) for 5 min and then deflated to induce reactive hyperaemia.

RH-PAT values were assessed at baseline and at 6 months. Endothelial dysfunction was defined as reactive hyperaemia index (RHI) <0.670. Previous studies have demonstrated that the RH-PAT technology has excellent reproducibility [15, 16].

### Derivatives of reactive oxygen metabolites (d-ROMs) evaluation

Oxidative stress is defined as an imbalance between the production of reactive oxygen metabolites (ROMs) and the removal of reactive oxygen species by a variety of endogenous and exogenous antioxidants. In the current study, we assessed oxidative stress using a simple method for the evaluation of ROMs, the recently developed d-ROMs test [17]. This assay is relatively inexpensive, can be performed in minutes, and has been used to assess the effectiveness of various antioxidant treatment strategies [18]. The d-ROMs test evaluates free radical activity by measuring serum levels of hydroperoxides (Diacron, Grosseto, Italy). The results of the d-ROMs test are expressed in arbitrary units, so-called Caratelli Units (U.CARR), where 1 U.CARR corresponds to 0.08 mg/100 ml H_2_O_2_ [19].

### Other measurements

Venous blood samples and urinary samples were taken in the morning after an overnight fast.

We evaluated the following parameters at baseline and after 12 and 24 weeks of treatment: HbA1c, eGFR, and urinary albumin excretion. HbA1c was measured using the automated analyzer “MetaboLead HbA1c” (KYOWA MEDEX CO., LTD., Tokyo, Japan). Serum low-density lipoprotein cholesterol and high-density lipoprotein cholesterol levels were also measured using standard enzymatic methods (MetaboLead HDL-C and MetaboLead LDL-C, KYOWA MEDEX CO., LTD., Tokyo, Japan). Serum triglyceride levels were measured using enzymatic assays (Wako Pure Chemical Industries, Ltd., Osaka). Urinary albumin excretion was measured by immunonephelometry using a kit from Roche Diagnostics (Rotkreuz, Switzerland). eGFR was calculated as 194 × serum creatinine^−1.094^ × age^−0.287^ in males, and as 194 × serum creatinine^−1.094^ × age^−0.287^ × 0.739 in females. L-FABP is a urinary biomarker of renal tubular injury and was measured by a two-step sandwich ELISA assay (LSI Medience Corporation, Tokyo, Japan). This biomarker has been evaluated previously in relation to diabetic kidney disease, but the results of different studies were conflicting [20–22]. The other oxidative stress markers, 8-hydroxy-2′-deoxyguanosine (8-OHdG) and 8-isoprostane, which were evaluated at baseline and 24 weeks, were measured by LSI Medience Corporation.

### Statistical analysis

Data are shown as mean ± standard deviation. Differences between continuous variables were analysed by the paired *t* test, unpaired *t* test, Mann–Whitney U test, and Wilcoxon’s matched pairs test as appropriate. Categorical variables were compared by the Chi square test. *P* values <0.05 were considered significant. All analyses were performed using Prism 6 (GraphPad Software, Inc., San Diego, CA, USA) or StatMate V (Nihon 3B Scientific Inc., Niigata, Japan).

## Results

**Fig. 2:**
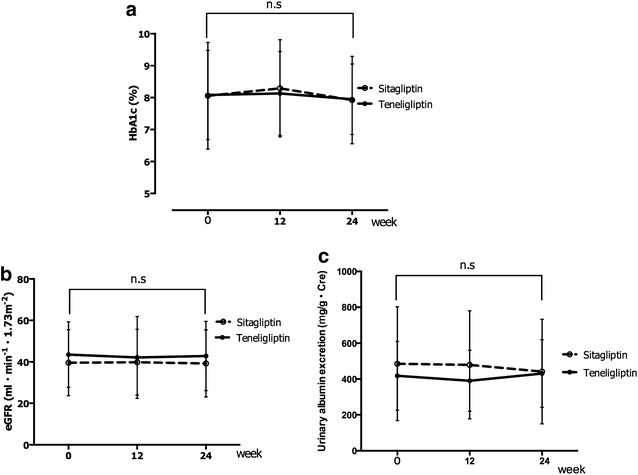
Changes in HbA1c (**a**), eGFR (**b**), and urinary albumin excretion (**c**). There were no significant differences in the levels of HbA1c, eGFR, and urinary albumin excretion between the sitagliptin and teneligliptin treatment groups

**Fig. 3:**
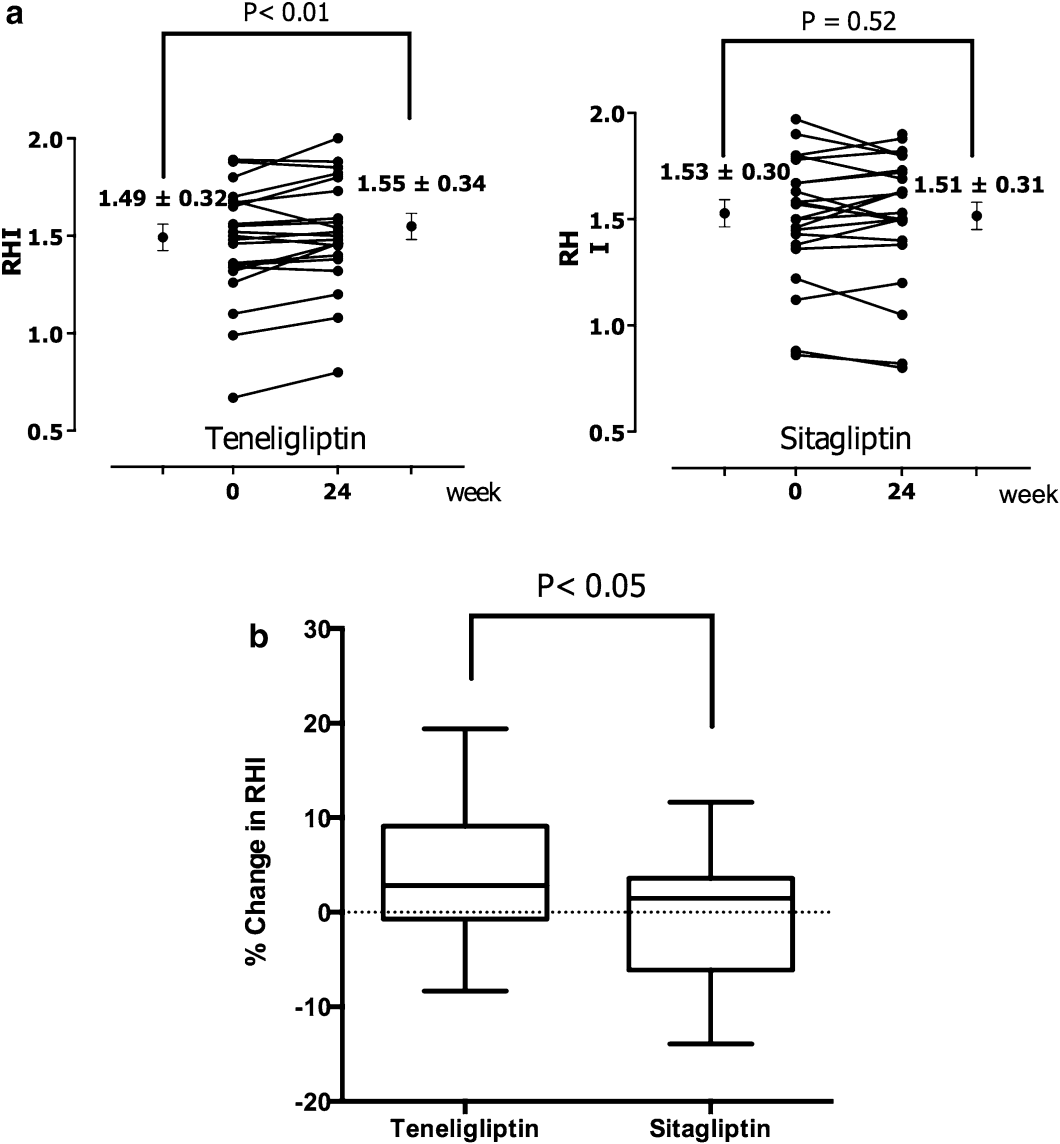
Changes in RHI in both groups (**a**) and the comparison of improvement in RHI (**b**). Percent change in RHI [RHI after 24 weeks—RHI before treatment]/RHI before treatment. RHI values significantly improved in the teneligliptin group; the percent change in RHI was also significantly greater in the teneligliptin group. *RHI* reactive hyperaemia index

**Fig. 4:**
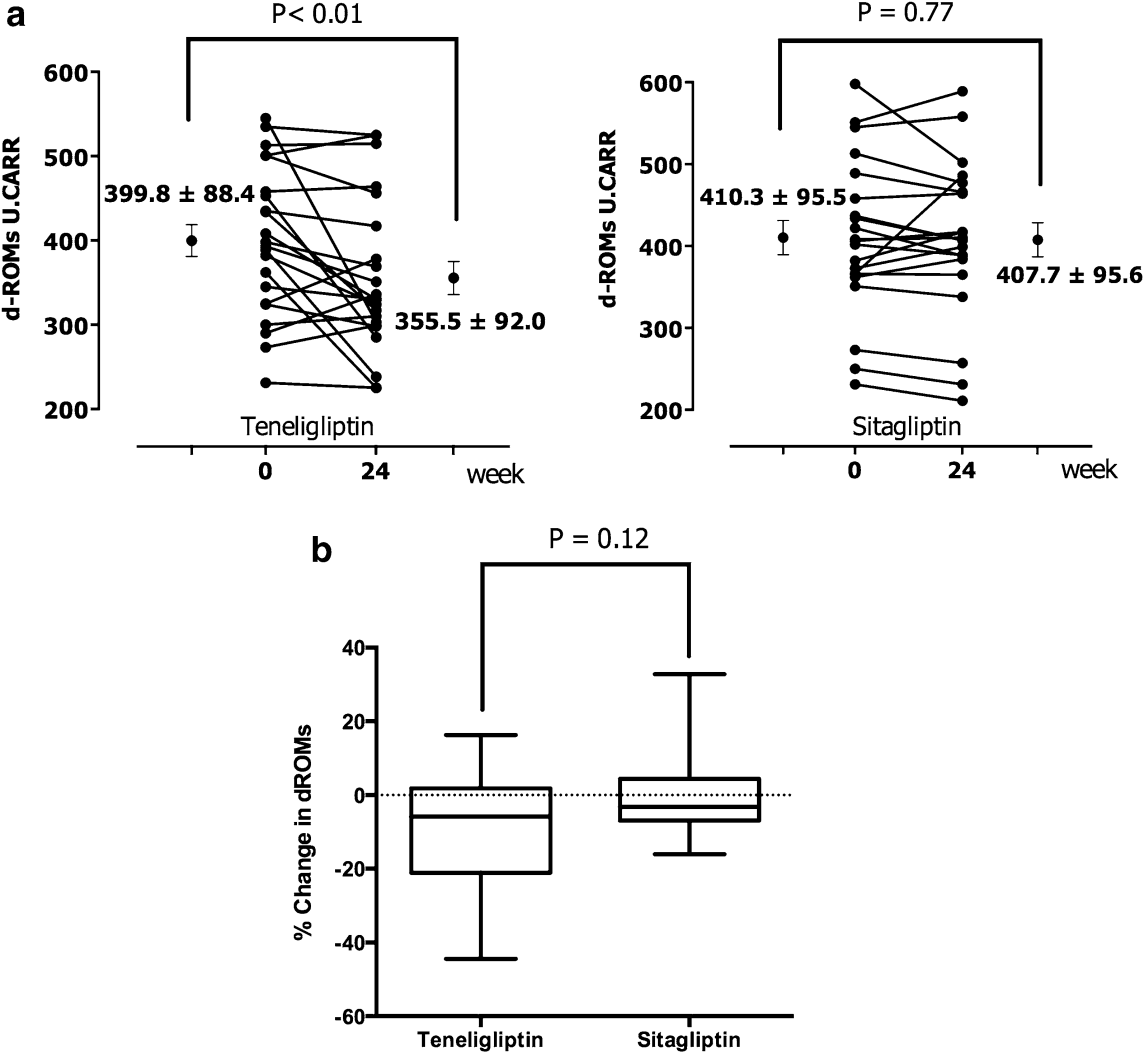
Changes in d-ROMs in both groups (**a**); comparison of percent change in d-ROMs (**b**). Percent change in d-ROMs: [d-ROMs after 24 weeks of treatment—d-ROMs before treatment]/baseline d-ROMs value. A biomarker of oxidative stress, d-ROMS, significantly decreased in the teneligliptin group. The percent change in d-ROMs in the teneligliptin group was slightly lower than that in the sitagliptin group

Patients’ clinical data are shown in Table 1. There were no significant differences between groups in any clinical or biochemical parameter, including the number of patients in each group with hypertension, being treated for hypertension, with dyslipidaemia, or being treated for dyslipidaemia at baseline. After the 24-weeek treatment period, there were no significant differences between the groups in the levels of HbA1c, eGFR, and (log) urinary albumin excretion (Fig. 2a–c). Moreover, fasting glucose levels, C-peptide, lipid profiles, and blood pressure did not differ significantly before and after treatment in either group (Table 2). RHI values significantly improved from 1.49 ± 0.32 to 1.55 ± 0.29 (*P* < 0.01) in the teneligliptin group, while in the sitagliptin group, RHI values did not differ significantly, from 1.50 ± 0.3 to 1.49 ± 0.31 (Fig. 3a). The percent change in RHI in the teneligliptin group was also significantly greater than that in the sitagliptin group (Fig. 3b, *P* < 0.05). d-ROMs, as a biomarker of oxidative stress, also decreased similarly in the teneligliptin group from 399.8 ± 88.4 to 355.5 ± 92.0 U.CARR (*P* < 0.01) (Fig. 4a). The percent change in d-ROMs in the teneligliptin group was slightly decreased compared to that in the sitagliptin group (Fig. 4b). Furthermore, 8-OHdG was significantly reduced at 24 weeks in the teneligliptin group from 7.1 ± 4.9 to 5.4 ± 2.9 ng/m Cre (Table 2; *P* < 0.05) compared with the sitagliptin group (6.4 ± 1.5 − 7.4 ± 2.1 ng/mg Cre). Urinary 8-isoprostane levels slightly increased in both groups at the end of the study, particularly in the sitagliptin group when compared to the levels in the teneligliptin group (Table 2; *P* < 0.05).

**Table 1:** Clinical characteristics of the subjects at baseline

	Teneligliptin	Sitagliptin	P value
Age (years)	70.0 ± 3.9	65.4 ± 10.7	0.912
Sex (M/F)	22 (10/12)	23 (12/11)	0.652
Body mass index (kg/m^2^)	22.1 ± 3.4	23.6 ± 1.9	0.209
HbA1c % (NGSP)	8.1 ± 1.4	8.0 ± 1.7	0.739
Duration of diabetes (years)	13.2 ± 3.0	12.8 ± 6.3	0.952
eGFR (mL/min/1.73m^2^)	43.5 ± 15.8	39.6 ± 15.9	0.394
Urinary albumin excretion (µg/g Cre)	418.4 (93.5–299.5)	485.0 (67.0–729.3)	0.750
Statins n (%)	16 (72.7 %)	17 (80.9 %)	0.627
ACEI or ARB n (%)	20 (90.1 %)	20 (95.2 %)	0.578
Biguanide n (%)	3 (13.6 %)	4 (19.0 %)	0.631
Sulfonylurea or glinide n (%)	7 (31.8 %)	9 (42.9 %)	0.454
Insulin n (%)	12 (54.5 %)	9 (42.9 %)	0.443

Data are mean ± SD or median and interquartile range

*ACEI* angiotensin-converting enzyme inhibitor, *ARB* angiotensin II receptor blocker

**Table 2:** Comparison of clinical and biochemical parameters at baseline and 24 weeks

	Teneligliptin (n = 22)		Sitagliptin (n = 23)		P^1^ value	P^2^ value
	Baseline	24 weeks	Baseline	24 weeks		
HbA1c % (NGSP)	8.1 ± 1.4	8.0 ± 1.2	8.0 ± 1.7	7.9 ± 1.4	0.27	0.39
Fasting glucose (mg/dL)	161.5 ± 49.7	157.7 ± 31.7	150.0 ± 26.3	161.7 ± 33.6	0.8	0.19
C-peptide (ng/mL)	1.8 ± 0.9	2.0 ± 0.6	2.5 ± 1.0	2.5 ± 1.1	0.32	0.33
SBP (mmHg)	133.3 ± 13.4	132.4 ± 14.2	139.1 ± 22.9	131.4 ± 16.8	0.79	0.06
DBP (mmHg)	76.1 ± 11.0	74.0 ± 10.9	79.3 ± 17.0	75.4 ± 12.8	0.5	0.07
LDL-C (mg/dL)	107.1 ± 21.3	105.3 ± 19.4	98.2 ± 21.5	98.7 ± 21.6	0.71	0.91
HDL-C (mg/dL)	53.4 ± 13.2	50.7 ± 12.0	55.5 ± 16.8	50.1 ± 9.1	0.12	0.06
TG (mg/dL)	120.3 ± 41.3	129.3 ± 60.1	156.1 ± 99.9	149.0 ± 70.2	0.51	0.61
eGFR (ml/min^−1^/1.73 m^2^)	43.5 ± 15.8	42.8 ± 16.7	39.6 ± 15.9	39.2 ± 16.2	0.36	0.77
Urinary albumin (mg/g Cre)	141.0 (93.5–299.5)	176.0 (84.5-268.0)	141.5 (67.0–729.3)	165.5 (52.8-546.0)	0.58	0.32
Log urinary albumin (mg/g Cre)	2.3 ± 0.5	2.3 ± 0.5	2.3 ± 0.6	2.3 ± 0.6	0.73	0.25
Urinary 8-OHdG (ng/mg Cre)	7.1 ± 4.9	5.4 ± 2.9	6.4 ± 1.5	7.4 ± 2.1	0.03*	0.1
Urinary L-FABP (µg/g Cre)	25.7 (6.3–118.0)	14.5 (7.0–64.9)	48.2 (7.2–91.9)	55.0 (9.6–110.8)	0.02*	0.22
Urinary 8-isoprostane (pg/mgCr)	199.0 (151.5–424.0)	297.0 (212.5–440.0)	258.0 (227.0–429.0)	353.0 (301.0–578.0)	0.06	0.01*

Data are mean ± SD or median and interquartile range

*SBP* systolic blood pressure, *DBP* diastolic blood pressure, *LDL*-*C* low-density lipoprotein cholesterol, *HDL* high-density lipoprotein cholesterol, *TG* triglycerides, *eGER* estimated glomerular filtration rate, *8*-*OHdG* 8-hydroxy-2′-deoxyguanosine, *L*-*FABP* liver-type fatty acid binding protein

* P^1^ value: <0.05, comparison of respective data between baseline and after 24 weeks treatment with teneligliptin

* P^2 ^value: <0.05, comparison of respective data between baseline and after 6 month treatment with sitagliptin

Baseline values and changes in urine markers of kidney injury (urinary L-FABP) are reported in Table 2. Compared with the starting period, teneligliptin, and not sitagliptin, was associated with a significant reduction of urinary L-FABP (*P* < 0.05), whereas urinary β2-microglobulin and N-acetyl b-d-glucosaminidase levels did not change in either group (data not shown).

**Fig. 5:**
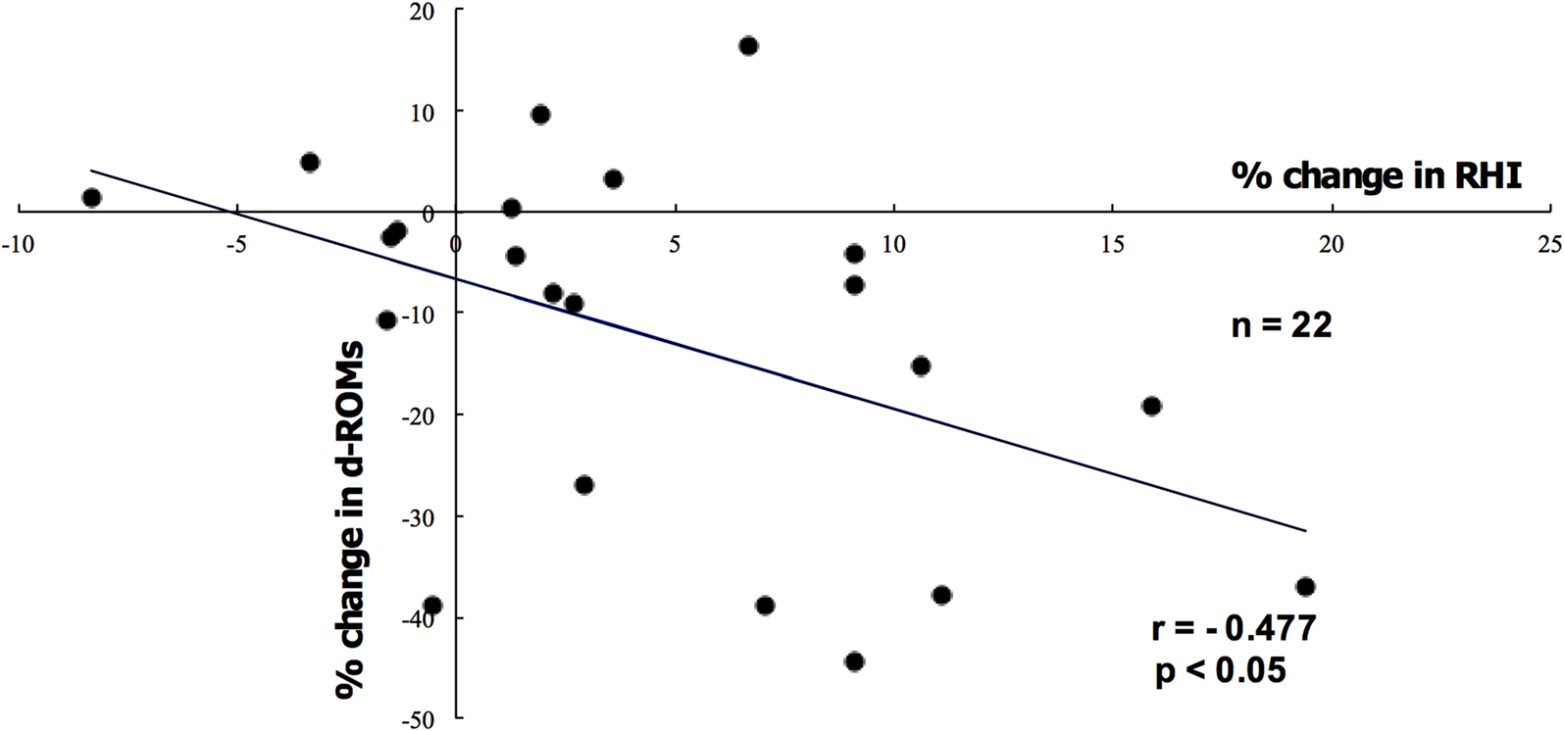
Correlation between percent change in RHI and percent change in d-ROMs in the teneligliptin group. The percent change in RHI strongly and negatively correlated with the percent change in d-ROMs in the teneligliptin group (n = 22)

Figure 5 shows the percent changes in RHI (the value at the starting of the trial minus that after 24 weeks divided by the starting value) upon treatment with teneligliptin, but not sitagliptin. The percent change in RHI strongly correlated negatively with the percent change in d-ROMs in the teneligliptin group (Fig. 5; *P* < 0.05).

## Discussion

The results of the present study showed that 24-week teneligliptin treatment improved RHI values, as demonstrated by the reduction in d-ROMs and urinary 8-OHdG levels, in patients with type 2 diabetes and CKD who had received sitagliptin for at least 12 months prior to switching to teneligliptin. In addition, we observed that teneligliptin treatment also reduced urinary L-FABP levels regardless of the reduction of urinary albumin excretion. These results suggest that the antioxidative and renoprotective effects of teneligliptin might be stronger than those of other DPP-4 inhibitors, particularly sitagliptin, and might be independent of its blood glucose-lowering effects.

Mori et al. reported that the DPP-4 inhibitor sitagliptin can prevent progression of diabetic nephropathy by reducing the severity of proteinuria and albuminuria [23]. However, the mechanism of DPP-4 inhibitor-induced improvement of albuminuria is unclear. In the present study, since the included patients had already received DPP-4 treatment for at least 12 months, urinary albumin excretion was not reduced in either the sitagliptin or the teneligliptin group. Notably, only switching to teneligliptin, and not sitagliptin continuation, reduced urinary L-FABP at 24 weeks.

Urinary L-FABP levels have been reported to associate with the histological severity of renal tubulointerstitial lesions in humans [21, 24]. Although urinary albumin excretion appears to reflect glomerular damage and subsequently renal tubulointerstitial damage, recently cases of diabetes have been reported in which renal function rapidly decreased without an increase in urinary albumin excretion [25]. Therefore, Araki et al. reported that urinary L-FABP might reflect tubulointerstitial damage and predict the progression of deteriorating renal function [21]. In addition, increasing urinary L-FABP levels were associated with deteriorating renal function and the high incidence of atherosclerotic cardiovascular disease (ASCVD) in type 2 diabetes. Thus, improvement of urinary L-FABP levels might be clinically useful for patients at high risk for renal disease and ASCVD. Urinary L-FABP is known to be a renoprotective protein localized predominantly in the proximal tubules, which has antioxidant properties and indicates the elevation of oxidative stress markers. One of the mechanisms responsible in part for the reduction of urinary L-FABP levels by teneligliptin treatment might be attenuation of oxidative stress.

In the present study, we also evaluated levels of urinary 8-OHdG to assess the effect of antioxidative stress on tubulointerstitial kidney damage. Urinary 8-OHdG levels were slightly increased in both treatment groups, whereas urinary 8-OHdG significantly increased from baseline values only in the sitagliptin group. Further long-term studies are needed for more precise evaluation of the effects of teneligliptin on kidney function compared with other DPP-4 inhibitors.

Previous studies have reported that RHI correlates with vascular endothelial dysfunction and is a risk factor for atherosclerosis and cardiovascular disease [15, 26]. Various researchers have reported RHI cutoff values of <1.82 [27], <1.70 [28] and <1.63 [29] in Japanese patients with type 2 diabetes at high risk of atherosclerotic cardiovascular disease. The normal range of RHI values still remains controversial. Therefore, in this present study, the change in RHI values might be an important factor reflecting endothelial function during teneligliptin treatment.

Endo-PAT is a noninvasive, quantitative, and repeatable technique that captures a beat-to-beat recording of the finger arterial pulse-wave amplitude with pneumatic probes. Therefore, RHI values can be used to evaluate vascular condition and treatment efficacy. According these previous reports, we used RHI values as a reproducible index of endothelial dysfunction. In the present study, teneligliptin treatment was associated with significant improvement of digitally recorded RHI values in type 2 diabetes patients with CKD. Clinical trials in patients with type 2 diabetes demonstrated that DPP-4 inhibitors improve endothelial function, as measured by reactive hyperaemia peripheral arterial tonometry and flow-mediated dilatation [16, 30]. However, in this study, we focused on type 2 diabetes patients with chronic kidney disease. We emphasize that this present study is the first report to evaluate the efficacy of teneligliptin on endothelial function and antioxidative effects of teneligliptin in tubulointerstitium and endothelium compared with sitagliptin in patients with type 2 diabetes and chronic kidney disease who are at high risk of ASCVD.

We recently reported that teneligliptin decreased the proportion of time in hyperglycaemia and increased the proportion of time at normal glucose levels in a 24-h period and significantly improved glucose fluctuations over 24 h [31]. By contrast, Nonaka et al. reported that 100 mg/day sitagliptin, but not 50 mg/day, provided substantial 24-h glucose lowering effects [32]. The differences between teneligliptin and sitagliptin observed in this study might be due to the fact that the dosage of sitagliptin used in Japan (25 mg/day) is much lower. Moreover, a recent study has shown that teneligliptin has a high potency for inhibiting DPP-4 activity compared with other DPP-4 inhibitors [33].

We also directly observed antioxidative effects of teneligliptin as demonstrated by decreased levels of d-ROMs, a novel global oxidative stress marker that is easy to measure. In addition, we showed that the reduction of d-ROMs levels by teneligliptin treatment was correlated with the amelioration of RHI values. These results suggest that this effect of teneligliptin might be associated with suppression of glucose fluctuation independently affecting renal function. Glucose fluctuation has been reported to cause endothelial dysfunction and oxidative stress generation more than stable high glucose in type 2 diabetes patients [34] and in vitro [35]. Rizzo et al. also reported that the suppression of glycaemic variability is associated with reduction of oxidative stress and markers of systemic inflammation in patients with type 2 diabetes [36]. Since our study focused on the influence of teneligliptin on oxidative stress markers and endothelial function, we measured fasting glucose level and C-peptide but did not examine postprandial glucose, glucose fluctuation, or inflammatory cytokines. However, we have already reported the improvement of glucose fluctuations caused by teneligliptin in Japanese patients with type 2 diabetes [31]. Furthermore, Kimura et al. recently demonstrated that a structural feature of teneligliptin, specifically having a sulphur atom within the molecule, was beneficial for radical scavenging. These beneficial effects of teneligliptin may lead to the reduction of oxidative stress [37]. Nevertheless, the present study has some limitations. First, the randomized clinical trial used open-label, not double-blind, administration of the study drug; however, the concealment of allocation and the use of an objective end-point assessment strengthened the significance of the results. Second, the number of participants was relatively small, and the study duration was short. Although we measured the expression of ICAM-1/VCAM-1 in both the sitagliptin and teneligliptin treatment groups, ICAM-1/VCAM-1 expression did not significantly decrease in either the teneligliptin or sitagliptin group. Nakagami et al. reported that long-term treatment with teneligliptin significantly decreased ICAM-1 expression in hypertensive rats and that this effect might be due, in part, to alleviating inflammation in the vascular system [38]. Longer-term clinical trials might be required to verify the effect of teneligliptin on the expression of ICAM-1/VCAM-1 in type 2 diabetes patients. However, we believe that the significant changes in some biomarkers, which indicate antioxidative effects, by teneligliptin treatment provide novel evidence of DPP-4 inhibitors. Third, the dosage of sitagliptin was relatively small, based on the proper dose for Japanese patients with type 2 diabetes and CKD. Fourth, we did not evaluate the relationship between glucose fluctuation and oxidative stress or RHI values.

Finally, we do not know whether the antioxidative effects of teneligliptin, as demonstrated in this study, could reduce cardiovascular morbidity in type 2 diabetes patients with CKD. The outcomes of trials of DPP-4 inhibitors, including sitagliptin, saxagliptin, and alogliptin, showed that these agents did not increase or decrease the number of major adverse cardiovascular events [39–42]. Additional information from future experimental studies, as well as clinical studies, with regard to teneligliptin is needed to address these results in patients with type 2 diabetes and CKD.

## Conclusions

In conclusion, the present study demonstrated that teneligliptin exhibits beneficial effects on both oxidative stress and endothelial function in Japanese patients with type 2 diabetes and CKD. These findings suggest that teneligliptin might be more useful than other DPP-4 inhibitors in the treatment of type 2 diabetes with CKD.

## Abbreviations

8-OHdG: 8-hydroxy-2′-deoxyguanosine
ASCVD: atherosclerotic cardiovascular disease
CKD: chronic kidney disease
DPP-4: dipeptidyl peptidase-4
eGFR: estimated glomerular filtration rate
HbA1c: haemoglobin A1c
L-FABP: liver-type fatty acid binding protein
PAT: peripheral arterial tonometry
RHI: reactive hyperaemia index
RH-PAT: reactive hyperaemia peripheral arterial tonometry
ROMs: reactive oxygen metabolites
